# Effect of an Integrated Case-based Nutrition Curriculum on Medical Education at Qazvin University of Medical Sciences, Iran

**DOI:** 10.5539/gjhs.v4n1p112

**Published:** 2012-01-01

**Authors:** Ahmad Afaghi, Ali Akbar Haj Agha Mohamadi, Ramin Sarchami, Amir Ziaee

**Affiliations:** Metabolic Diseases Research Center Qazvin University of Medical Sciences, Qazvin, Iran; Metabolic Diseases Research Center Qazvin University of Medical Sciences, Qazvin, Iran Email: ah43867@yahoo.com; Education Development Center Qazvin University of Medical Sciences, Qazvin, Iran; Metabolic Diseases Research Center Qazvin University of Medical Sciences, Qazvin, Iran

**Keywords:** Integrated education, Nutrition curriculum, Perception

## Abstract

**Introduction:**

Nutrition education is identified as an important part of medical education by organizations. Qazvin University of Medical Sciences, school of medicine (QUMS SOM), has a required basic nutrition course of 36 hr in second year of medical school, but education experts reports show that the course does not provide required therapeutic skills for graduate student.

**Method:**

We decided to organize an 8-hr diet therapy work shop in order to develop a patient experience clinical based case study teaching to enhance clinical skills at QUMS SOM.

**Results:**

Students’ perception score about adequacy of nutrition instruction increased from 20% (at baseline) to 70% (after intervention). The mean nutrition knowledge score of total students in clinical nutrition were different between before and one month after integration (8.3±2.5, 13.4±3.2, *P* < 0.001). And two groups of participants including staggers and interns had similar nutritional knowledge score at pre-test (7.9±2.6 and 8.9±2.3 respectively).

**Conclusion:**

Implemented nutrition curriculum at QUMS was appropriate method to enhance student’s perception about nutrition integration and to increase and translate the knowledge to clinical practice.

## 1. Introduction

Nutrition training for medical education and its effect on health and the prevention of disease is identified as an important part of medical education by organizations such as American Society for Clinical Nutrition (ASCN) (1990), the American Medical Student Association (AMSA) (1996), and the National Academy of Science (NAS) (1985). Challenges to include more nutrition education in the medical schools education program backs to decades ago (Stare, 1949; Shills, 1994; Dutra-de-Oliveira and Marchini, 1995; Rasmann-Nuhlicek *et al.*, 1995). These efforts have had variable success, and nutrition education is continually being discussed as a subject in need of medical students (McLaren, 1994; Weinsier, 1995; Gershoff, 1996). Diet has a main role in the pathogenesis of diabetes, cancer, stroke, osteoporosis, heart disease, liver disease, and other obesity related disease, however most medical students report that they do not get enough nutritional training during medical school (Lo, 2000). In spite of the minimum medical nutrition education, 25 hr, recommended by NAS (1985), many medical school still do not provide the recommended minimum hr of nutrition education. And those who used to offer nutrition course of 27 hr in first or second year of medical education were enable to provide competency of basic therapeutic applications of foods and diets (Lo, 2000; Woods, 2006). Different approaches in integrated nutrition curriculums on medical education have been performed. At Arizona University, School of Medicine, the implementation of nutrition curriculum resulted in doubling of the total hours of required instruction (35 compared with 75 h) in prospect courses throughout the 4 y of undergraduate medical studies. The objective structured clinical examination (OSCE) score in nutrition significantly improved after implementation of curriculum (41.7% compared with 50.6%) and students perception of inadequacy of medical-nutrition training decreased (68.4% compared with 11.5%) (Taren *et al.*, 2001). In integration, at Tufs University, School of Medicine, development of standardized patient cases in nutrition counseling for cardiovascular disease and weight loss and its incorporation into the clerkship and residency programs in internal medicine and family medicine was focused. This procedure was along with expansion of nutrition education including slide show in nutrition topics such as clinical cases, dietary analysis, and patients’ handouts. Also nutrition sessions and patient experience included in the education program. This expanded nutrition program in internal and family medicine along with the standardized patient experience resulted in an excellent rating from physicians, residents, and medical students (Woods, 2006).

Qazvin University of Medical Sciences, school of medicine (QUMS SOM), with a total of 7 years education program, has a required basic nutrition course of 36 hr in second year of medical school, but education experts reports show that the course does not provide required therapeutic skills for graduate student. The nutrition course currently is running in year 2 of basic science during which medical students are not involved in clinical setting and they do not know how to apply their learned materials in practice. Therefore we decided to organize an 8-hr diet therapy work shop in order to develop a patient experience clinical based case study teaching to enhance clinical skills regarding the role of nutrition in chronic disease, metabolic, heart, renal, intestinal disease, assessment of current dietary intake, and weight management at QUMS SOM. Also we aimed to increase clinical nutrition skill of students to arrange an objective structured clinical examination (OSCE) and to improve its score over time.

## 2. Subjects and Methodes

### 2.1 Subjects

The nutrition curriculum described in this report was implemented at the QUMS COM. The project was initiated in May of 2011 and forty six medical students (22-25 years) who were involved in 4-weeks community medicine training, enrolled in the project. There was no conflict of interest for students to participate. The project was approved by QUMS SOM.

### 2.2 Methods

For the development and implementation of the integrated nutrition curriculum at QUMS SOM we developed a new curriculum. In addition to baseline 36 hr single basic nutrition course which normally is offered in year 2 of medical school education, we organized an 8-hr diet therapy work shop. Then we developed a patient experience clinical based case study teaching, to enhance clinical skills regarding the role of nutrition in chronic disease, metabolic, heart, renal, and intestinal disease; and assessment of current dietary intake and weight management. The curriculum initiates during 1 month of students’ community medicine training (year 4-5), and is intended the 30 cases of patients having different diet pattern to be practiced over time throughout year 5, 6, and 7

### 2.3 Nutrition curriculum

For developing new nutrition curriculum, we conducted a self-developed, 25 multiple choice nutrition therapy questionnaire (total 25 score) to assess medical students’ knowledge. At the beginning of the performed pre test, we sought the students’ perception about nutrition instruction at baseline. The question was:

Do you believe that the time devoted to your instruction in nutrition was?

a) inadequate
b) appropriate
c) excessive
d) and also any extra explanation

We were aware, most medical students at QUMS were not satisfied with nutrition course. We were interested to observe whether the proposed nutrition curriculum at QUMS would result in a more favorable response. In other word, would nutrition workshop and also exposure to the curriculum result in either reduced percentage of students reporting that inadequate time was devoted to instruction in nutrition or an increased percentage of students reporting that appropriate time was devoted to instruction in nutrition? This change in perception will be comparable with responses to similar question in post-test, one month after conducting nutrition workshop.

For organizing the diet therapy workshop, the content of an 8-hr case-based, problem oriented tutorial curriculum design workshop reviewed by a nutritional professional and an internal medicine specialist. Next, a meeting was held with the 5 faculty members who actively were teaching the content of workshop, to determine the nutrition objectives and the potentials for expanding the nutrition content. Last, case-based, problem oriented clinical approach teaching implemented and the new nutrition content integrated into the clinical course. This integrated approach allows the nutritional knowledge of the curriculum to be taught and practice throughout the medical education.

### 2.4 Assessment of outcome measures

Assessment of the project included 3 primary outcomes. The first evaluation included the measurement of knowledge of student in the nutrition therapy area at the end of one month community medicine training. The second evaluation technique is supposed to measure applied skill in diet therapy using the OSCE. The third evaluation method will use the post graduation questionnaire for student evaluation of curriculum nutrition content.

### 2.5 Objective Structured Clinical Examination

The OSCE is a clinical approach in medical education to evaluate clinical skills among medical students. This multi station format evaluation tools uses standardized, trained patients to assess the performance of a wide range of clinical skills including applied nutrition skills.

### 2.6 Statistical analysis

To evaluate the nutrition curriculum the questions were asked: 1) Do medical student improve their applied nutrition knowledge after conducted nutrition workshop. 2) Do students apply learned nutrition knowledge in a clinical setting? 3) Do performance on the OSCE nutrition items and its score improve in relation to exposure to the nutrition curriculum overtime? All statistical analysis for this research was conducted with use of SPSS 16 statistical software (SPPSS Inc, Chicago). To address the first question, the statistical analysis (paired t-test) included a comparison of the scores of the medical students before (pre-test before nutrition workshop) and after implementation of the nutrition curriculum (post-test after nutrition workshop).

Performance on the OSCE is the second focus for outcome analysis. To address the second question, we will compare the post-test scores with nutrition subscale score of the first conducted OSCE using paired t-test. To determine whether the nutrition OSCE scores are improving over time, statistical analysis will be conducted among the students of different years after implementation of nutritional curriculum.

### 2.7 The pilot study

A pilot study was completed prior to the main study on a six internship medical student. The student passed all the procedure of the project including attending clinical nutrition workshop and nutrition clinic for training. Comparison of pre and post test scores and clinical skill evaluation showed a significant improvement in nutrition knowledge and applied nutrition in clinical setting. Theses students were not part of the actual data collection

## 3. Results

### 3.1 Nutrition content

The nutrition content before and after the integrated nutrition curriculum is demonstrated in table 1. At the start of the integrated nutrition curriculum in May 2011, there were 34h (2 credits unit) of required nutrition instruction during semester 2 of year 2 of medical education. After the integrated nutrition curriculum, 8 hr extra nutrition instruction in relation to diet therapy included in medical education.

**Table 1:** Hours of nutrition curriculum content by course at baseline and after the development and implementation of the integrated nutrition curriculum at the Qazvin University of Medical Science School of Medicine

Nutrition instruction at baseline (h)		Extra medical nutrition therapy instruction after implementation of the nutrition curriculum (h)	
Carbohydrates, sources and related disease	2	gastrointestinal tract disorder	1/2
Protein, sources and related malnutrition	2	Liver disorder & Encephalopathy	1/2
Fat, sources	2	Diabetes mellitus	1/2
Energy, measurement of energy	2	Anemia	1/2
Minerals	2	Heart disease	1/2
Vitamins	2	Renal disorder	1/2
Food groups, food habits and meal planning	2	Infection and diarrhea	1/2
Maternal nutrition	2	Weight management program	1/2
Nutrition during childhood, nutrition in aging	2	Weight gain program	1/2
Nutrition assessment	2	Gluten intolerance	1/2
Anemia (iron and folic acid deficiency)	2	Diet in gout	1/2
Food safety and food poisoning	2	Diet during pregnancy, lactation	1/2
Nutrition obesity and heart disease	2	Food groups and exchange list application in diabetes	1/2
Breast feeding and infants	2	Glycemic index of foods and its application	1/2
Nutrition during pregnancy and lactation	2	Complementary medicine	1/2
Nutrition assessment, PEM	2	Nutrition assessment of patients	1/2
Malnutrition in Iran and around world	2	Vitamins and minerals sources	1/2
Total hr	34		8

### 3.2 Nutrition knowledge and OSCE score

In pre-test 20% of the students reported that the time devoted to nutrition was adequate. The students also explained in open question that, they suggest the “Clinical Nutrition” to be offered during years 4, 5, and 6 of their clinical practice education. In post-test 70% of students indicated that the nutrition instruction was adequate. Also, in open answer question, they expressed that they are satisfied with current ongoing program. Paired t-test showed that the mean nutrition knowledge score of total students in applied nutrition were different between before and one month after integration (8.3±2.5, 13.4±3.2, *P* < 0.001) (table 2). And two groups of participants including staggers and interns had similar nutritional knowledge score at pre-test (7.9±2.6 and 8.9±2.3 respectively). Comparing the score of OSCE with nutrition knowledge and also comparing OSCE scores over time in ongoing integration will be reported separately in due time.

**Table 2:** Students’ perception and knowledge score in pre and post-test

	Pre-test	Post-test	*P*
Perception about adequacy of nutrition course hours	20%	75%	
Knowledge score			
Stager (n = 26)	7.9±2.6	13.2±3.9	< 0.001
Internship (n = 20)	8.9±2.3	13.7±2.1	< 0.001
Total students (n = 46)	8.3±2.5	13.4±3.2	< 0.001

## 4. Discussion

Our primary evaluation of the nutrition education program indicated that the integrated curriculum was an appropriate and time-efficient model for integrating clinical dietetic into medical education. This approach resulted in significant increase in medical students diet therapy knowledge (8.3±2.5, 13.4±3.2, respectively *P* < 0.001) and enhancing student-reported perception of the adequacy of nutrition education in new implemented program. Observing similar nutritional knowledge score at pre-test among two groups of staggers and interns students (7.9±2.6 and 8.9±2.3 respectively) revealed that the traditional approach in medical education does not significantly improve clinical nutrition knowledge during medical education. This ongoing nutrition education program introduces a clinical orientation to basic nutrition and diet therapy and provides repeated exposure to nutrition information that enhances clinical skill over time. Medical students often do not know how to apply learned nutrition knowledge. The case-based, student-organized learning is appropriate solution to this problem. An important aspect of the evaluation is that how students will be able to use their knowledge and skills in a clinical setting as will be assessed by the OSCE. Previous researches have shown the effectiveness of the OSCE in evaluating the using knowledge and skills gained during medical education to future clinical practice (Rutala *et al.*, 1992; Dupras and Li, 1995). OSCE score is similar to traditional methods in evaluating general ability and is also able to evaluate clinical ability in standardized manner (Merrick *et al.*, 2000). There are other approaches to nutrition curriculum in medical education. In the nutrition integration program at University of Arizona, College of Medicine program, the curriculum does not comprise a single nutrition course but rather integrated nutrition content into required courses throughout the 4 yr of undergraduate medical studies (Taren *et al.*, 2001). We believe that integrating cased-based clinical dietetic allows students to identify patients and medical conditions that would benefit from nutrition intervention for disease prevention, diet therapy, and appropriate nutrition prescriptions and referrals. Our integration program provides totally 42 hr nutrition education which is more than minimum 25 hr recommended by NAS in 1985 (1985). This nutrition education prepares medical students to improves and enhance their medical nutrition skill in clinics during patient managements throughout their clinical practice.

There are limitations for conducting the project. Physicians in clinical settings need to cooperate with integration process. For the supporting the project of integrating nutrition in clinical medical education, it is necessary the clinical nutrition topics and nutrition knowledge take a prominent place on students final and national board examination as has been discussed in existing literature (Hark *et al.*, 1997). The final internship exams needs to move away from multiple-choice content questions, turning instead toward more case-based clinical nutrition problem-solving questions

## 5. Conclusion

In conclusion, the case based nutrition curriculum at QUMS is cost-effective and appropriate method to translate the knowledge to clinical practice. The nutrition curriculum implemented at the QUMS which resulted in improvement of knowledge and also more favorable response to the adequacy of time devoted to instruction in nutrition, needs to be replicated in other medical school for improvement of suggested nutrition integration.
